# Plasma MicroRNA-21 Predicts Postoperative Pulmonary Complications in Patients Undergoing Pneumoresection

**DOI:** 10.1155/2016/3591934

**Published:** 2016-05-12

**Authors:** Yaling Liu, Peiying Li, Xinyu Cheng, Weifeng Yu, Liqun Yang, Hui Zhu

**Affiliations:** Renji Hospital, Shanghai Jiaotong University School of Medicine, Shanghai 200127, China

## Abstract

Postoperative pulmonary complication (PPC) remains the most common postoperative complication in patients undergoing noncardiac thoracic surgery. We conducted the clinical study to determine the diagnostic role of miRNA-21 in noncardiac thoracic surgery. 368 patients undergoing noncardiac thoracic surgery were recruited. Blood samples were collected before anesthesia and 2 hours after incision during surgery for RT-PCR measurement of miRNA-21. PPC occurrence, extrapulmonary complications, duration of ICU stay, and death within 1 year were evaluated. The overall rate of PPCs following surgery was 10.32%. A high relative miRNA-21 level was an independent risk factor for PPCs within 7 days (OR, 2.69; 95% CI, 1.25–5.66; and *P* < 0.001). High miRNA-21 was also associated with an increased risk of extrapulmonary complications (OR, 3.62; 95% CI, 2.26–5.81; and *P* < 0.001), prolonged ICU stay (OR, 6.54; 95% CI, 2.26–18.19; and *P* < 0.001), increased death within 30 days (OR, 6.17; 95% CI, 2.11–18.08; and *P* < 0.001), and death within 1 year (OR, 7.30; 95% CI, 2.76–19.28; and *P* < 0.001). In summary, plasma miRNA-21 may serve as a novel biomarker of PPCs for patients undergoing noncardiac thoracic surgery.

## 1. Introduction

Postoperative pulmonary complication (PPC) is one of the major causes of mortality and morbidity in the intensive care unit (ICU). PPC frequently occurs in patients undergoing noncardiac thoracic surgery [1, 2]. The occurrence of some pulmonary issues may be delayed, even up to 2-3 weeks following surgery, leading to unexpected readmission to the hospital or even death. It was recently reported that the most common reasons for readmission are pulmonary issues, which account for 29% of total readmissions in patients undergoing esophageal cancer surgeries [3]. However, reliable predictors of PPCs are still lacking in clinical practice. Berry et al. [4] showed that even preoperative pulmonary function tests do not predict PPCs after lobectomy.

MicroRNAs (miRNAs) are small noncoding RNA molecules of 21 to 24 nucleotides that regulate gene expression by targeting the 3′-untranslated regions of RNA transcripts and downregulating or silencing target mRNA, resulting in the termination of translation. The roles of miRNAs in the regulation of all major cellular functions, including cell proliferation, differentiation, metabolism, and apoptosis, are being increasingly recognized [5]. Recent studies have identified miRNAs as critical modulators of lung and systemic inflammation via the regulation of cell responses to TGF-*α* signaling [5–9]. Importantly, some miRNAs can be detected in the peripheral blood, rendering them promising novel biomarkers for inflammatory status and the prognosis of cardiovascular diseases, especially coronary artery disease [10, 11].

miRNA-21 plays a crucial role in a plethora of biological functions and diseases, including immunological and developmental processes, cancer, cardiovascular diseases, and pulmonary inflammation. Recently, additional roles of miRNA-21 in cardiovascular and pulmonary diseases, including cardiac and pulmonary fibrosis as well as myocardial infarction, have been described [12]. Due to the critical functions of its target proteins in various signaling pathways, miRNA-21 has become an attractive target for genetic and pharmacological modulation in various disease conditions.

In particular, a number of studies link miRNA-21 with pulmonary functions and inflammation. For example, it is specifically expressed in acute respiratory distress syndrome (ARDS) [13]. Lu et al. [14] demonstrated that, in allergic airway inflammation, miRNA-21 modulates the Th cell immune process. Sawant et al. [15] found that miRNA-21 is elevated in the sera of both asthma and eosinophilic esophagitis patients. Levels of miRNA-21 are significantly higher in asthmatic children than children without asthma [16]. It is commonly noted that inflammatory stimuli induce miRNA-21, which plays an important role in modulating acute inflammation [14, 17–19]. miRNA-21 is correlated with C-reactive protein and fibrinogen levels, suggesting that it is a biomarker of inflammation [20]. Qi et al. [21] found that miR-21 is upregulated during an LPS challenge and the serum miRNA-21 concentration is elevated in ARDS patients compared with healthy volunteers. In the resolution of wound inflammation, elevated macrophage miRNA-21 promotes efferocytosis and silences the target genes* PTEN* and* PDCD4*, which results in a net anti-inflammatory phenotype [22]. Accordingly, miRNA-21 plays an important role in inflammatory reactions. However, it is not clear whether variation in the plasma miRNA-21 concentration is related to noncardiac thoracic surgery and the occurrence of PPCs.

In the present study, we determined the association between plasma miRNA-21 levels and PPCs in patients undergoing noncardiac thoracic surgery. The early detection or prediction of PPCs may benefit patients by influencing the therapeutic strategy or prompting the initiation of anti-inflammatory treatments to prevent the occurrence of pulmonary complications and improve the overall outcomes of lung surgery patients.

## 2. Materials and Methods

### 2.1. Study Design and Subjects

This prospective study was conducted at Renji Hospital, Shanghai Jiaotong University School of Medicine. The study protocol and statistical analyses were approved by the Shanghai Jiaotong University Ethics Committee and adhered to the principles of the Declaration of Helsinki. This study was registered in the Chinese Clinical Trial Registry (ChiCTR-OOB-15006767). Written informed consent was obtained from the designated surrogate of each patient on the day before surgery.

Inclusion criteria were as follows: patients older than 18 years of age and scheduled for elective noncardiac thoracic surgery, including pulmonary lobectomy, segmentectomy, and pulmonary wedge resection. Patients were excluded if they had mechanical ventilation within 2 weeks before surgery, had a body mass index (BMI) of 35 or higher, or had a history of respiratory failure, heart failure, or sepsis within 2 weeks before surgery. Patients who developed respiratory failure (PO_2_ < 60 mmHg and PCO_2_ < 50 mmHg) after surgery were also excluded.

### 2.2. Blood Sampling

Using ethylene diamine tetraacetic acid (EDTA), venous blood samples (5 mL) from a central vein were obtained at baseline (before anesthesia) and at two hours after incision during surgery. These samples were immediately centrifuged at 3,000 to 4,000 rpm for 15 minutes. Plasma was frozen at −80°C until subsequent analysis.

### 2.3. Data Collection

Demographics and potential confounding factors with respect to PPC development were recorded, including age, smoking status, American Anesthesia Association (ASA) score, BMI, history of chronic obstructive pulmonary disease (COPD), diabetes, hypertension, and chronic heart disease.

### 2.4. Measurements of miRNA Expression

#### 2.4.1. RNA Isolation

RNA was isolated using a TRIzol-based miRNA isolation protocol (TRIzol BD from Sigma; St. Louis, MO, USA). Owing to high phenol contamination in these samples, additional isopropanol or ammonium precipitation steps were included. In addition, the miRNeasy Kit (Qiagen, Hilden, Germany) was used, which combines phenol/guanidine-based lysis of samples and silica membrane-based purification of total RNA (>18 nucleotides) in combination with the blood derivate-specific TRIzol BD from Sigma. The optimized protocol using the Qiagen miRNA Kit was then used for all subsequent studies with 250 *μ*L of EDTA plasma.

#### 2.4.2. Detection and Quantification of miRNAs by Quantitative PCR

RNA was obtained as described above and diluted 1 : 10. Diluted RNA (5 *μ*L) was reverse transcribed using the TaqMan miRNA Reverse Transcription Kit (ABI, Waltham, MA, USA) according to the manufacturer's instructions. Subsequently, 3 *μ*L of the product was used to detect miRNA expression by quantitative PCR using TaqMan MicroRNA Assay Kits (ABI) for the corresponding miRNA [23]. As previously reported [24], samples were supplemented with 5 nmol/L* Caenorhabditis elegans* miRNA-39 (cel-miRNA-39) for normalization. The miRNA values were calculated as 2^−(CT[miRNA]−CT[cel-miRNA-39])^. The internal control was U6. The miRNA-21 expression levels are reported as both absolute and relative values. The relative expression level was estimated as the ratio between miRNA-21 levels at 2 hours after incision during surgery to the baseline before surgery.

### 2.5. Clinical Endpoints

The primary endpoints were the incidence of PPCs [25] and ARDS. PPCs were defined as the occurrence of any of the following complications within 7 days after surgery: microatelectasis, bronchospasm, hypoxemia, atelectasis, hypercarbia, pleural effusion, pneumonia, and respiratory failure. The details of this definition are shown in Table 1. ARDS was defined according to the most recent consensus [26].

Secondary outcomes included extrapulmonary complications occurring within 7 days after surgery, which were defined as sepsis, severe sepsis, and septic shock (defined according to consensus criteria) [27], duration of ICU stay, and rate of death from any cause 30 days and 1 year after surgery.

### 2.6. Statistical Analyses

The statistical analysis was performed using GraphPad Prism version 5.0 (GraphPad Software, La Jolla, CA, USA). Continuous variables are expressed as means ± SD. Categorical variables are expressed as frequencies and percentages, and a 2-tailed *χ* ^2^ or Fisher's exact test was used to assess difference between groups. The normality of the distribution of miRNA-21 values was analyzed with the Kolmogorov-Smirnov test.

Univariate associations between potential predictor variables and PPCs were investigated using logistic regression modeling. All variables that were significant at a nominal 2-tailed *P* ≤ 0.05 were included in multivariable logistic models using a combination stepwise selection method. Results are displayed as odds ratios and 95% confidence intervals (CI).

## 3. Results

### 3.1. Study Population

**Figure 1:**
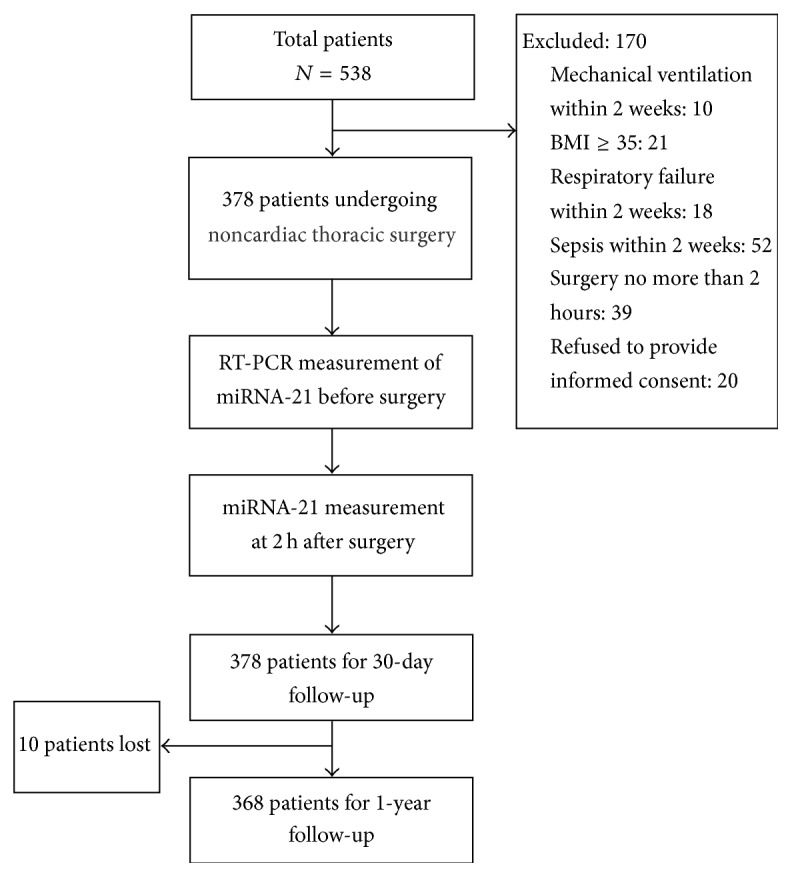
Study design and flow chart. Totally 538 patients were recruited and 170 were excluded. Ten patients were lost during 1-year follow-up. Ultimately 368 patients finished the study.

From January 21, 2012, through December 10, 2013, 538 patients scheduled to undergo lung surgery were recruited in the study, and 170 patients were excluded. A total of 368 patients undergoing noncardiac thoracic surgery were finally included in the study and were followed for 30 days and 1 year after surgery (Figure 1). Ten patients were lost during the follow-up period. Among all patients, 47.6% were male. The mean age was 59.7 ± 5.8 years old, and mean BMI was 26.1 ± 8.5. A total of 193 (52.5%) patients had a history of COPD and 196 (53.3%) were smokers or ex-smokers for 6 weeks. Patients' characteristics are shown in Table 2.

### 3.2. Clinical Characteristics of Patients with and without PPCs

The characteristics of patients with and without PPCs are summarized in Table 3. The extensiveness of the surgery did not affect the outcome. The patient characteristics for the two groups were comparable, except for one-lung ventilation (OLV), which was frequent in the PPC group than in the non-PPC group (68.4% versus 47.6%, *P* = 0.02).

### 3.3. Distribution of Clinical Outcomes and Their Relationship with miRNA-21

The data for primary and secondary clinical endpoints were collected and analyzed by univariate logistic regression with respect to miRNA-21. There were 6 deaths at the one-year follow-up, of which 4 patients died from septic shock and 2 patients from respiratory failure. PPCs, ARDS, extrapulmonary complications, ICU stay, and death were significantly related to miRNA-21 fold change before and 2 h after surgery (Table 4).

### 3.4. Potential Risk Factors of PPCs Identified by Univariate and Multivariate Analyses

Using a univariate logistic regression analysis, we found that patients with high miRNA-21 fold changes (OR, 4.58; 95% CI, 3.16–6.87; *P* < 0.001) and OLV (OR, 2.39; 95% CI, 1.17–4.89; *P* < 0.001) were more likely to develop PPCs within 7 days after surgery (Table 5).

**Figure 2:**
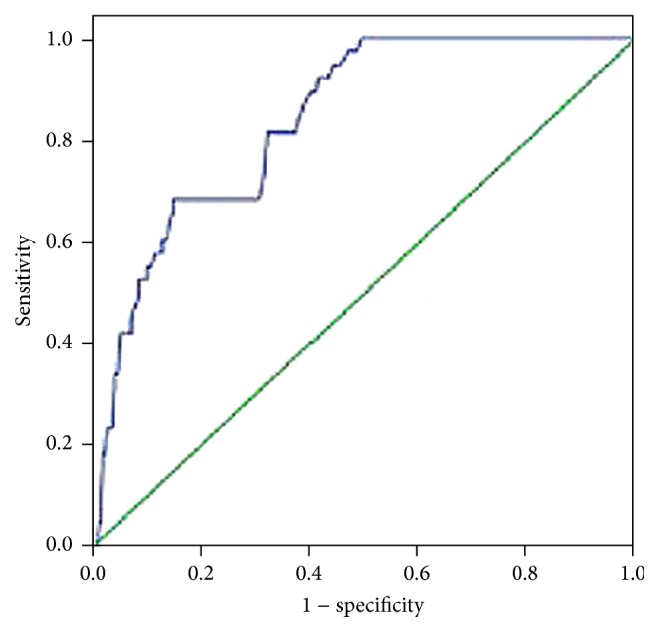
In a receiver operative characteristic (ROC) curve analysis, the area under the curve (AUC) of miRNA-21 levels during surgery for PPCs was 0.838 (95% CI, 0.782–0.894; *P* < 0.05). At a cutoff value of 1.745, miRNA-21 had a sensitivity of 68.4% and a specificity of 70.6% for predicting PPCs within 7 days after surgery.

Based on the Kolmogorov-Smirnov test, the miRNA-21 values were normally distributed (*P* > 0.05). To further validate whether factors identified in the univariate analyses were associated with the development of PPCs, we performed a multivariate logistic regression analysis. PPC was associated with not only the miRNA-21 fold change (OR, 2.69; 95% CI, 1.25–5.66; *P* < 0.001), but also OLV (OR, 1.88; 95% CI, 1.02–3.57; *P* < 0.001) (Table 5). In a receiver operative characteristic curve analysis, the area under the curve (AUC) of miRNA-21 during surgery for PPCs was 0.84 (95% CI, 0.78–0.90; *P* < 0.05), as shown in Figure 2. At a cutoff value of 1.745 (calculated from the AUC of maximizing the sensitivity and specificity), miRNA-21 had a sensitivity of 68.4% and a specificity of 70.6% for predicting PPCs within 7 days after surgery. An increase in miRNA-21 served as an indicator of PPCs.

### 3.5. MicroRNA-21 Expression Detection and Its Association with PPC Development in Patients Undergoing Noncardiac Thoracic Surgery

We estimated the average miRNA-21 expression at baseline and 2 hours after surgery in PPC and non-PPC patients. In the PPC group, miRNA-21 expression increased significantly (386.47 ± 29.87 versus 238.42 ± 20.30, *P* < 0.01). In contrast, in the non-PPC group, the average miRNA-21 levels did not change obviously (276.52 ± 30.68 versus 241.65 ± 33.96, *P* = 0.57) (Table 6).

### 3.6. OLV as a Predictor of PPCs during Noncardiac Thoracic Surgery

As summarized in Table 3, OLV was associated with increased PPCs (68.4% versus 47.6, *P* = 0.02). In univariate and multivariate logistic regression analyses, OLV had a relationship with PPCs (OR, 2.39; CI, 1.17–4.89; *P* < 0.001 and OR, 1.88; 95% CI, 1.02–3.57; *P* < 0.001, resp.).

## 4. Discussion

Using RT-PCR, we measured miRNA-21 levels in the plasma of patients undergoing noncardiac thoracic surgery. We found that high relative miRNA-21 levels predicted not only PPCs within 7 days, but also extrapulmonary systemic complications, such as sepsis, septic shock, prolonged ICU stay, and death within 30 days and 1 year. To the best of our knowledge, this is the first study identifying plasma miRNA-21 as a reliable predictor of PPCs as well as extrapulmonary postoperative complications in patients undergoing noncardiac thoracic surgery.

Emerging evidence from animal studies indicates that miRNAs are important mediators of inflammatory responses, including lung inflammatory processes. For instance, in a LPS- and ventilation-induced lung injury model, miRNA-146a is involved in controlling toll-like receptor and cytokine signaling [23]. Additionally, miRNA-21 is upregulated in many inflammatory states, including the inflamed lung in LPS-treated mice [28] and allergic airway inflammation [14–16]. We measured plasma levels of miRNA-21 expression in ARDS based on previous reports demonstrating an association [13].

Several studies have focused on plasma miRNAs as promising novel biomarkers for inflammatory status [20–22] and the prognosis of cardiovascular diseases, especially coronary artery disease [10, 11]. Das et al. [22] found that miRNA-21 accounted for an anti-inflammatory phenotype. The discrepancy between proinflammatory and anti-inflammatory characteristics with respect to miRNA-21 may be attributed to organ and tissue specificity.

Most of the PPCs defined in this study, such as pneumonia, atelectasis, pleural effusion, and bronchospasm, are associated with excessive inflammatory responses characterized by the infiltration of inflammatory cells and overproduction of inflammatory mediators, which contributes to the pathogenesis of lung injury [29]. Therefore, these conditions can be reasonably detected by plasma miRNA-21, which is considered an indicator of pulmonary inflammation [28]. Hypoxemia, hypercarbia, and respiratory failure are essentially consequences of severe inflammatory PPCs; thus, hypoxia or respiratory failure may occur in patients suffering from increased pulmonary inflammation with high levels of plasma miRNA-21. High plasma miRNA-21 levels were also able to predict the occurrence of extrapulmonary complications, increased ICU stays, and death within 30 days and even 1 year. This suggests that patients with high miRNA-21 levels who developed PPCs following lung surgery might further develop sepsis and septic shock and require prolonged ICU stays, potentially resulting in death. Another explanation for these results is that this study failed to recruit a sufficient sample of patients with extrapulmonary complications who did not die from these complications.

Another predictor of PPCs and extrapulmonary postoperative complications is OLV. In the present study, OLV had an obvious association with clinical endpoints. OLV induces lung injury, which is related to volutrauma, atelectrauma, biotrauma, atelectasis, hyperperfusion, oxidation, and ischemia-reperfusion injury [30]. These mechanisms result in adverse clinical outcomes.

Plasma biomarkers have potentially important clinical implications. The clinical applicability of miRNA-21 as a biomarker is promising. Plasma samples are easily accessible. Measurements of miRNA-21 are feasible and inexpensive. Laboratories have the equipment necessary for such an analysis. Results will be obtained in 30 minutes. The current cost in China is 30–40 US dollars. In addition, assaying miRNA-21 in the plasma is minimally invasive. We believe that it is possible to generalize this approach to predict PPCs and ARDS in noncardiac thoracic surgeries at other hospitals.

This study had several important limitations. The high odds ratios and wide 95% CI indicated that the sample size was insufficient. Another limitation is that the current study population had a relatively high prevalence of COPD, hypertension, and diabetes mellitus. Despite high rates of comorbidities for patients recruited in this study, the incidence of PPCs was relatively lower (10.3%) than previous reports, which showed that 21.7% patients undergoing lobectomy by thoracotomy and 17.8% patients undergoing thoracoscopy developed PPCs [4, 31]. The discrepancy between the incidence of PPCs in our study and those of previous studies may also be attributed to the limited sample size in our study. We utilized a single set of data for our analysis; accordingly, the relationship between miRNA-21 and PPCs needs to be validated in an independent study.

Additional studies with larger sample sizes and wider inclusion criteria are warranted to confirm the use of plasma miRNA-21 as a convenient and novel biomarker for the early prediction of PPCs in patients undergoing noncardiac thoracic surgery.

## 5. Conclusions

An increase in plasma miRNA-21 during noncardiac thoracic surgery predicts the occurrence of PPCs within 7 days following surgery, extrapulmonary complications, death with 30 days following surgery, and prolonged ICU stay. Plasma miRNA-21 may serve as a novel biomarker for the early detection of PPCs.

## Figures and Tables

**Table 1:** Diagnosis of postoperative pulmonary complications.

Grade	Description
0	No pulmonary complications

1	Coughing without sputum Microatelectasis: no other special pathological reasons; with pulmonary symptoms and body temperature > 37.5°C; chest imaging can be normal Dyspnea: no other special pathological reasons

2	Cough and expectoration: no other special pathological reasons Bronchospasm: newly developed asthma or asthma exacerbations need to be treated Hypoxemia: SpO_2_ < 90% or PaO_2_ < 60 mmHg without oxygen Atelectasis: confirmed by chest X-ray with or without abnormal pulmonary symptoms or body temperature > 37.5°C Hypercapnia: temporary or requires treatment with medication or excessive ventilation

3	Pleural effusion: which requires drainage Suspected pneumonia: confirmed by chest X-ray with or without bacteriological evidence Diagnosed pneumonia: confirmed by chest X-ray and bacteriological evidence of Gram stain or culture Pneumothorax: X-ray showed chest air and no blood vessel bed: with or without dyspnea, chest pain, irritation, cough, and other symptoms Noninvasive mechanical ventilation or reintubation with invasive mechanical ventilation < 48 h

4	Respiratory failure: noninvasive mechanical ventilation or reintubation with invasive mechanical ventilation > 48 h

Complication grade is defined by the most severe description.

**Table 2:** Patient baseline characteristics.

Preoperative characteristics	Total (*n* = 368)
Age (years)	59.7 ± 5.8
Male (%)	175 (47.6)
BMI	26.1 ± 8.5
Current smoker (%)	196 (53.3)
COPD (%)	193 (52.5)
Hypertension (%)	180 (48.9)
Heart failure (%)	168 (45.7)
Diabetes (%)	188 (51.1)

Continuous data are presented as means ± SD; numerical data are presented as numbers (percentage among total patients). BMI: body mass index; COPD: chronic obstructive pulmonary disease.

**Table 3:** Clinical characteristics in patients with and without PPCs.

Variable	No PPCs (*n* = 330)	PPCs (*n* = 38)	*P* value
Age	59.7 ± 5.8	59.6 ± 6.3	0.88
Male (%)	47.3	50	0.75
BMI (mean ± SD)	26.1 ± 2.4	26.3 ± 2.3	0.71
Current smoker (%)	52.1	63.1	0.19
COPD (%)	53.3	39.5	0.11
Hypertension (%)	47.6	60.5	0.13
Heart failure (%)	47.3	31.6	0.06
Diabetes (%)	49.7	63.2	0.12
Surgical procedures (%)			
VATS pulmonary lobectomy	10.3	10.5	0.78
VATS segmentectomy	13.9	13.2	0.85
VATS pulmonary wedge resection	21.8	15.8	0.09
Pulmonary lobectomy	26.4	36.8	0.23
Segmentectomy	11.5	13.2	0.78
Pulmonary wedge resection	16.1	10.5	0.26
Operation time: means ± SD (min)	149.9 ± 36.3	160.9 ± 43.6	0.11
Blood loss: mean ± SD (mL)	107.3 ± 27.6	115.8 ± 21.4	0.94
OLV (%)	47.6	68.4	0.02

Continuous data are presented as means ± SD. Numerical data are presented as percentage among designated patient groups. *P* values were derived using 2-sample Student's *t*-test or Wilcoxon rank-sum test for continuous variables and 2-tailed *χ* ^2^ or Fisher's exact test for categorical variables. OLV: one-lung ventilation.

**Table 4:** Distribution of primary and secondary endpoints during 1-year follow-up and their relationships with miRNA-21 fold changes based on univariate logistic regression.

Category	*n* (%) or mean ± SD	OR	95% CI	*P* value
PPCs	38 (10.3)	4.58	3.16–6.87	<0.001
Microatelectasis	3 (0.8)			
Bronchospasm	10 (2.7)			
Hypoxemia	4 (1.0)			
Atelectasis	6 (1.6)			
Hypercarbia	5 (1.4)			
Pleural effusion	5 (1.4)			
Pneumonia	3 (0.8)			
Respiratory failure	2 (0.5)			
ARDS	6 (1.6)	1.56	1.15–4.87	0.03
Extrapulmonary complications	20 (5.4)	3.62	2.26–5.81	<0.001
Sepsis	8 (2.2)			
Severe sepsis	8 (2.2)			
Septic shock	4 (1.1)			
ICU stay	7.0 ± 3.2	6.54	2.26–18.19	<0.001
Death in 30 days	3 (0.8)	6.17	2.11–18.08	<0.001
Death in 1 year	6 (1.6)	7.30	2.76–19.28	<0.001

PPC: postoperative pulmonary complication; ARDS: acute respiratory distress syndrome; ICU: intensive care unit.

**Table 5:** Univariate and multivariate logistic regression of the relationships between MiRNA-21, OLV, and PPCs.

	Univariate			Multivariate		
	Odds ratio	95% CI	*P* value	Odds ratio	95% CI	*P* value
miRNA-21 fold change higher than 1.745	4.58	3.16–6.87	<0.001	2.69	1.25–5.66	<0.001
OLV	2.39	1.17–4.89	<0.001	1.88	1.02–3.57	<0.001

OLV: one-lung ventilation; PPC: postoperative pulmonary complication.

**Table 6:** miRNA-21 expression level according to PPCs.

miRNA-21	Before surgery	2 h after surgery	*P* value
With PPC	238.42 ± 20.30	386.47 ± 29.87	<0.01
Without PPC	241.65 ± 33.96	276.52 ± 30.68	0.57

PPC: postoperative pulmonary complication.

## Additional Points

(1) Postoperative pulmonary complication is a major cause of mortality after noncardiac thoracic surgery and there is no reliable biomarker to predict clinical prognosis. (2) The miR-21 is specifically expressed in acute respiratory distress syndrome. Inflammation plays an important role in pneumoresection and noncardiac thoracic surgery; accordingly, we speculate that miR-21 may be a marker of postoperative pulmonary complications. (3) miRNA-21 not only predicted the occurrence of PPCs and extrapulmonary complications, but was also a prognostic factor for death within 1 year. Patients undergoing one-lung ventilation during surgery were more likely to develop PPCs within 7 days after surgery. (4) MicroRNAs, especially miRNA-21, and one-lung ventilation in general anesthesia are closely related to noncardiac thoracic surgery outcomes.
